# Advances in Understanding of Structural Reorganization in the Hypothalamic Neurosecretory System

**DOI:** 10.3389/fendo.2017.00275

**Published:** 2017-10-17

**Authors:** Seiji Miyata

**Affiliations:** ^1^Department of Applied Biology, The Center for Advanced Insect Research Promotion (CAIRP), Kyoto Institute of Technology, Kyoto, Japan

**Keywords:** angiogenesis, gliogenesis, hypothalamus, neurosecretion, pituitary gland

## Abstract

The hypothalamic neurosecretory system synthesizes neuropeptides in hypothalamic nuclei and releases them from axonal terminals into the circulation in the neurohypophysis (NH) and median eminence (ME). This system plays a crucial role in regulating body fluid homeostasis and social behaviors as well as reproduction, growth, metabolism, and stress responses, and activity-dependent structural reorganization has been reported. Current knowledge on dynamic structural reorganization in the NH and ME, in which the axonal terminals of neurosecretory neurons directly contact the basement membrane (BM) of a fenestrated vasculature, is discussed herein. Glial cells, pituicytes in the NH and tanycytes in the ME, engulf axonal terminals and interpose their cellular processes between axonal terminals and the BM when hormonal demands are low. Increasing demands for neurosecretion result in the retraction of the cellular processes of glial cells from axonal terminals and the BM, permitting increased neurovascular contact. The shape conversion of pituicytes and tanycytes is mediated by neurotransmitters and sex steroid hormones, respectively. The NH and ME have a rough vascular BM profile of wide perivascular spaces and specialized extension structures called “perivascular protrusions.” Perivascular protrusions, the insides of which are occupied by the cellular processes of vascular mural cells pericytes, contribute to increasing neurovascular contact and, thus, the efficient diffusion of hypothalamic neuropeptides. A chronic physiological stimulation has been shown to increase perivascular protrusions *via* the shape conversion of pericytes and the profile of the vascular surface. Continuous angiogenesis occurs in the NH and ME of healthy normal adult rodents depending on the signaling of vascular endothelial growth factor (VEGF). The inhibition of VEGF signaling suppresses the proliferation of endothelial cells (ECs) and promotes their apoptosis, which results in decreases in the population of ECs and axonal terminals. Pituicytes and tanycytes are continuously replaced by the proliferation and differentiation of stem/progenitor cells, which may be regulated by matching those of ECs and axonal terminals. In conclusion, structural reorganization in the NH and ME is caused by the activity-dependent shape conversion of glial cells and vascular mural cells as well as the proliferation of endothelial and glial cells by angiogenesis and gliogenesis, respectively.

## General Introduction

**Figure 1:**
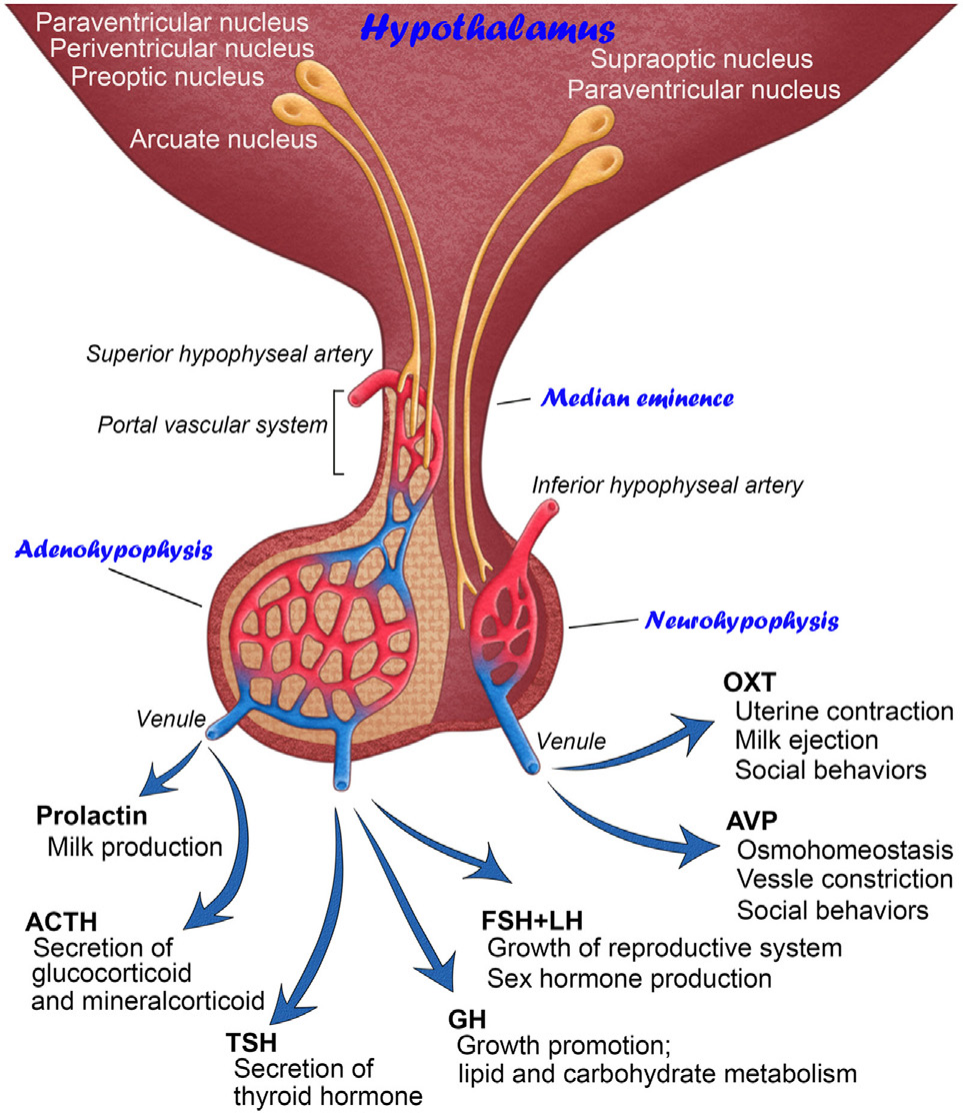
Schematic illustration showing the anatomy and functions of mammalian hypothalamic–neurohypophysial and hypothalamic–adenohypophyseal systems.

The pituitary gland is known as the “master gland” in mammals due to its function as the central endocrine regulator for fluid homeostasis, growth, reproduction, metabolism, and stress responses. Neurosecretion is defined as the synthesis and storage of neuropeptides in brain neurons and their release from axonal terminals into the circulation. Neurosecretory cells resemble non-neural endocrine cells in their actions; they release hormones into the circulation and regulate a number of physiological responses. Scharrer was the first to report a hypothalamic–pituitary neurosecretory system that exhibits similar secretory activity to that observed in endocrine cells of the peripheral system (1). These hypothalamic neurons conduct electrical impulses, similar to general brain neurons, but produce neuropeptides that are released into the circulation, unlike general brain neurons (2). There are currently two well-established hypothalamic–pituitary neurosecretory systems (Figure 1).

The hypothalamic–neurohypophysial system consists of magnocellular neurosecretory neurons that synthesize oxytocin (OXT) and arginine vasopressin (AVP) at somata in the hypothalamic supraoptic (SON) and paraventricular nuclei (PVN) and secrete these neuropeptides into the circulation from axonal terminals in the neurohypophysis (NH) (2). OXT is classically known to play a vital role in facilitating a range of physiological functions, such as labor induction and lactation (3). However, recent studies have shown more functions for OXT in social cognition, including emotion recognition, trust, and intersubjective selectivity (4, 5). The primary functions of AVP are the maintenance of body fluid homeostasis or proper osmolality *via* water reabsorption in the kidneys and the constriction of blood vessels (3). However, recent evidence indicates that AVP is also involved in a wide range of social behaviors (6). The deposition of tau protein was previously shown to be increased in the NH of aged humans, indicating that abnormal tau protein accumulation in magnocellular axons in older individuals may cause a dysfunction in body fluid homeostasis (7). Reductions in AVP-regulated aquaporins, renal urea, and sodium transporters with aging may result in multiple abnormalities in several physiological systems (8). Thus, the hypothalamic–neurohypophysial system plays vital roles for body fluid homeostasis and reproduction and is important for controlling social behaviors, and disturbances in this system lead to several homeostatic (9) and neuropsychiatric (10) dysfunctions.

The other type of hypothalamic–pituitary neurosecretory system is the hypothalamic–adenohypophyseal system (11). The hypothalamic–adenohypophyseal system, comprising neurosecretory neurons in the arcuate nucleus, preoptic area, periventricular nucleus, and ventromedial hypothalamus, synthesizes adenohypophyseal hormone-releasing factors and secretes them into the hypophyseal portal vein in the median eminence (ME) in order to control the secretion of adenohypophyseal hormones (12, 13). The adenohypophysis contains five types of endocrine cells: growth hormone (GH)-, prolactin-, gonadotropin-releasing hormone (GnRH)-, adrenocorticotropic hormone-, and thyroid-stimulating hormone-secreting cells. Adenohypophyseal hormones are known to be involved in many physiological regulatory systems, such as growth, reproduction, metabolism, and stress responses (14).

The present review introduces advances in structural reorganization in the NH and ME of adult mammals, in which axonal terminals directly contact the basement membrane (BM) of fenestrated capillaries. The following topics are discussed in this review: (1) activity-dependent neurovascular plastic events provided by glial cells and pericytes and (2) the role of angiogenic factors in shaping endothelial and glial cell populations.

## Size-Limited Vascular Permeability

The brain vasculature is generally characterized by a blood–brain barrier (BBB), which prevents the free entry of a number of bioactive and/or toxic molecules into the parenchyma of the brain (15). The BBB is important for maintaining the normal physiology of the brain and safety of neuronal tissues and a disturbance in the BBB leads to severe brain damage (16). However, the vasculature of the circumventricular organs (CVOs) lacks a typical BBB and possesses a fenestrated characterization unlike that in most of the other brain regions (17). CVOs are classified into two categories based on their main functions. Sensory CVOs, consisting of the organum vasculosum of the lamina terminalis, subfornical organ, and area postrema, monitor hormones, ions, osmolality, and pH in the blood and are endowed with a wide spectrum of receptors for blood-derived molecules (18). They integrate and transmit blood-derived information to other hypothalamic and extra-hypothalamic regions in order to mainly control body fluid and thermal homeostasis and inflammation (18, 19). The functions of sensory CVOs have already been described in detail (17). Secretory CVOs consisting of the NH and ME release hypothalamic neuropeptides as described above.

The BBB is defined as an endothelial cellular sheet that is endowed with tight and adherens junctions, which prevent the free entry of water-soluble molecules into the brain parenchyma (15, 20, 21). Alternatively, ECs express many kinds of transporter proteins on the luminal side of the EC membrane, which allow for the direct incorporation of amino acids, vitamins, hormones, proteins, or other compounds of the blood (22). However, the vasculature lacks the expression of tight junction proteins on ECs in the NH (23) and ME (24–26). Since there are no neuronal somata in the NH and the external zone of the ME, these regions do not need an EC barrier to protect neurons and, hence, their capillaries possess fenestrated features.

Blood-derived low-molecular-weight (LMW) molecules have been detected at the interstitial space after the administration of LMW tracers such as neutral amino acid alpha-aminoisobutyric acid [MW = 103 (27)], fluorescein isothiocyanate [FITC; MM = 390 (23, 28)], Evans blue [MM = 961 (28)], and Dextran 3,000 [MW = 3,000 (23)] in the NH; fluorescein [MM = 332 (29)], FITC (28, 30, 31), and Dextran 3,000 (31) in the external zone of the ME. The molecular weights of these tracers mimic those of most hypothalamic neuropeptides. In contrast to LMW molecules, blood-derived high-molecular-weight (HMW) molecules were not detected at the interstitial space after the administration of tracers, such as bovine serum albumin [MW = 70,000 (28)], Dextran 10,000 [MM = 10,000 (23)], and Dextran 70,000 [MM = 70,000 (28)] in the NH; immunoglobulin [MW = 150,000 (29)], bovine serum albumin [MW = 70,000 (28)], Dextran 10,000 [MW = 10,000 (31)], and Dextran 70,000 [MW = 70,000 (28)] at the interstitial space in the external zone of the ME.

This size-limited high vascular permeability is reasonable when considering the molecular weight of neurohypophysial and adenohypophyseal hormone-releasing hormones: the molecular weights of adenohypophyseal hormone-releasing hormones range from thyrotropin-releasing hormone (MW = 362.4) to growth hormone-releasing hormone (MW = 5,040.4), while those of neurohypophysial neuropeptides are OXT (MW = 1,007) and AVP (MW = 1,084). LMW-soluble molecules (molecular radius <3 nm) have been suggested to move passively through endothelial intercellular clefts in capillaries lacking the endothelial BBB. On the other hand, the transcellular pathway is considered to mediate the transport of blood-derived HMW-soluble proteins (molecular radius >3 nm) by caveolae *via* receptor-mediated, trans-endothelial channels or a fluid-phase pathway (32, 33). Horseradish peroxidase has the ability to penetrate the inner BM and EC layer and extensively accumulates between the inner and outer BM in the NH and ME, but some reaction products of horseradish peroxidase are also seen at the interstitial space (34). Horseradish peroxidase and germ agglutinin lectin are known to be incorporated by mannose receptor-mediated transcellular routes (35, 36). Thus, some, but not all, of HMW-soluble proteins are permeable to fenestrated capillaries in the ME and NH by a specific transport system.

**Figure 2:**
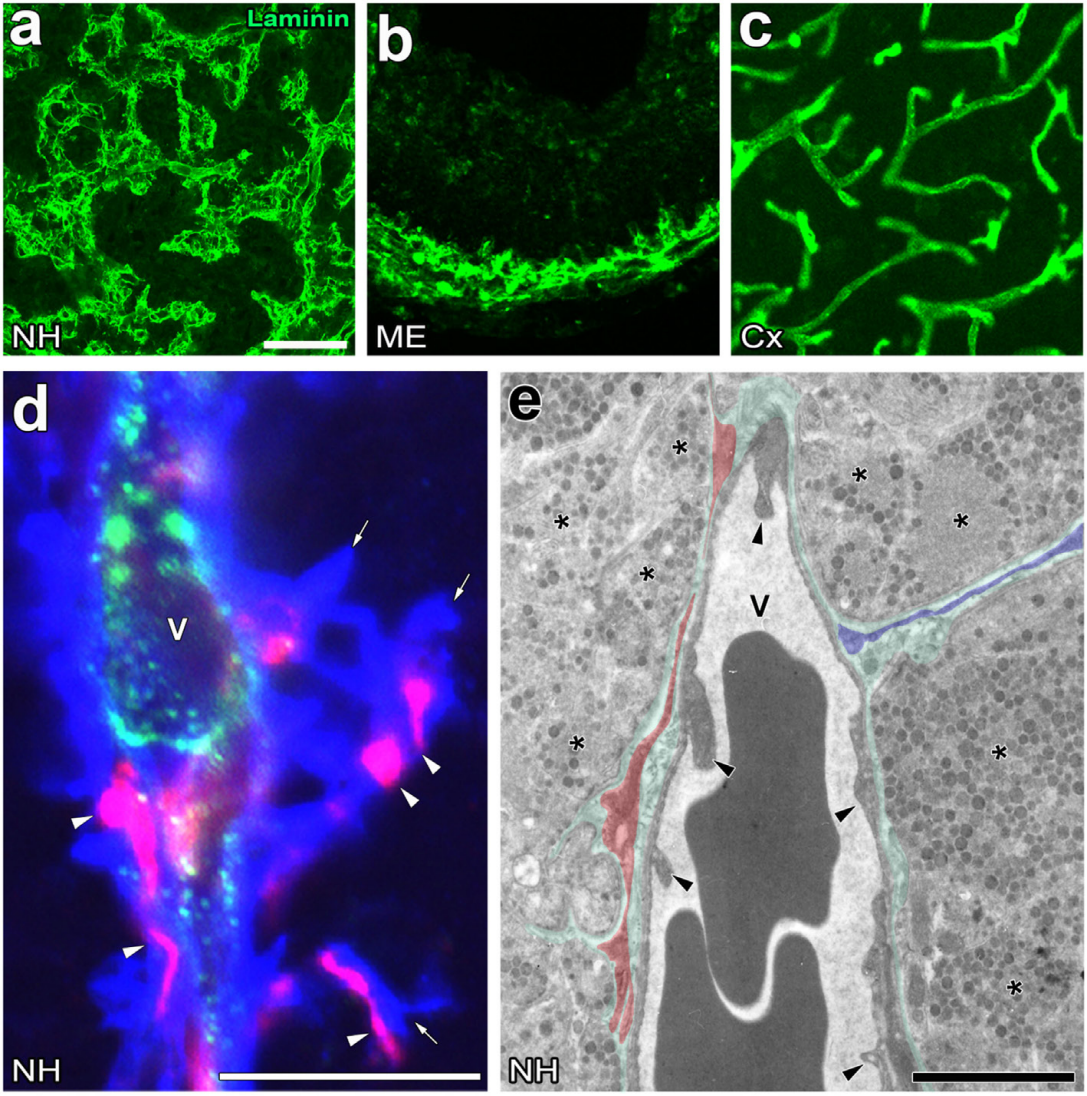
Unique perivascular structure with a wide perivascular space and perivascular protrusions in the neurohypophysis (NH) and median eminence (ME) of the adult mouse. Low magnification views show that the vasculature contour of the NH and ME is rough and complex, whereas that of the cerebral cortex is smooth **(A–C)**. High magnification views of triple labeling immunohistochemistry reveal the presence of a wide laminin-positive perivascular space and perivascular protrusions (open arrows), the inside of which contains desmin-positive pericytes (open arrowheads) **(D)**. An electron micrograph shows that pericytes typically localize in parallel with the inner basement membrane (BM, red shadow), whereas pericytes sometimes extend their cellular processes vertically with the inner BM to constitute perivascular protrusions (blue shadow) **(E)**. Green shadow and solid arrowheads indicate the perivascular space and endothelial cells, respectively. V, vascular lumen. Scale bars represent 50 μm **(A)**, 10 μm **(D)**, and 1 μm **(E)**. Micrographs are rearranged with courtesy from BioScientifica [**(A)** (40)] and permission from Springer [**(C)** (41)] and John Wiley & Sons [**(B)** (42)]; [**(D,E)** (23)].

The expression of collagen I is stronger at the inner BM than at the outer BM, whereas that of laminin is stronger at the outer BM than at the inner BM in these regions (23, 28). Moreover, the inner and outer BMs are both thicker in these neurosecretory brain regions than in other typical brain regions [(23); Figure 2]. In the glomerulus of the kidney, a thick BM exists between the fenestrated EC layer of the glomerular capillary side and the foot processes of podocytes of the urinary-space side (37). The glomerular BM is mainly composed of collagen IV, laminins, nidogen, and sulfated proteoglycans and contributes to the filtration barrier of the kidney (38). Mutations in genes encoding a subtype of either laminin or collagen type IV result in proteinuria, which often progresses to nephrotic syndrome and renal failure (37). In a previous study using conditional laminin γ1 knockout mice as well as adenovirus-mediated astrocyte-specific laminin knockdown mice, a lack of astrocytic laminin was shown to cause the breakdown of the BBB (39). Moreover, the lack of astrocytic laminin was found to affect astrocytic endfeet polarity, pericyte differentiation, and endothelial tight junction protein expression. Thus, it is probable that the outer BM has the ability to restrict diffusion of blood-derived molecules.

Plasmalemmal vesicle-1 (PV-1) is strongly expressed at ECs in the ME (43) and NH (23). PV-1 forms caveolae rings that surround lymphocytes and facilitate their transcellular migration across the EC layer (44). The expression of PV-1 increases when the BBB is disrupted, such as brain ischemia or brain tumors (45). A previous study reported that the inhibition of PV-1 expression attenuated vascular endothelial growth factor-A (VEGF-A)-induced caveola formation and increased the permeability of the 70-kDa HMW tracer molecule in the retinal vasculature containing the blood–retinal barrier, whereas its inhibition had no effect on the leakage of LMW fluorescein (46). Thus, PV-1 may control the transport of HMW molecules *via* an endothelial transcellular or caveolar pathway in neurosecretory regions.

## Significance of the Perivascular Space in Neurosecretion

**Figure 3:**
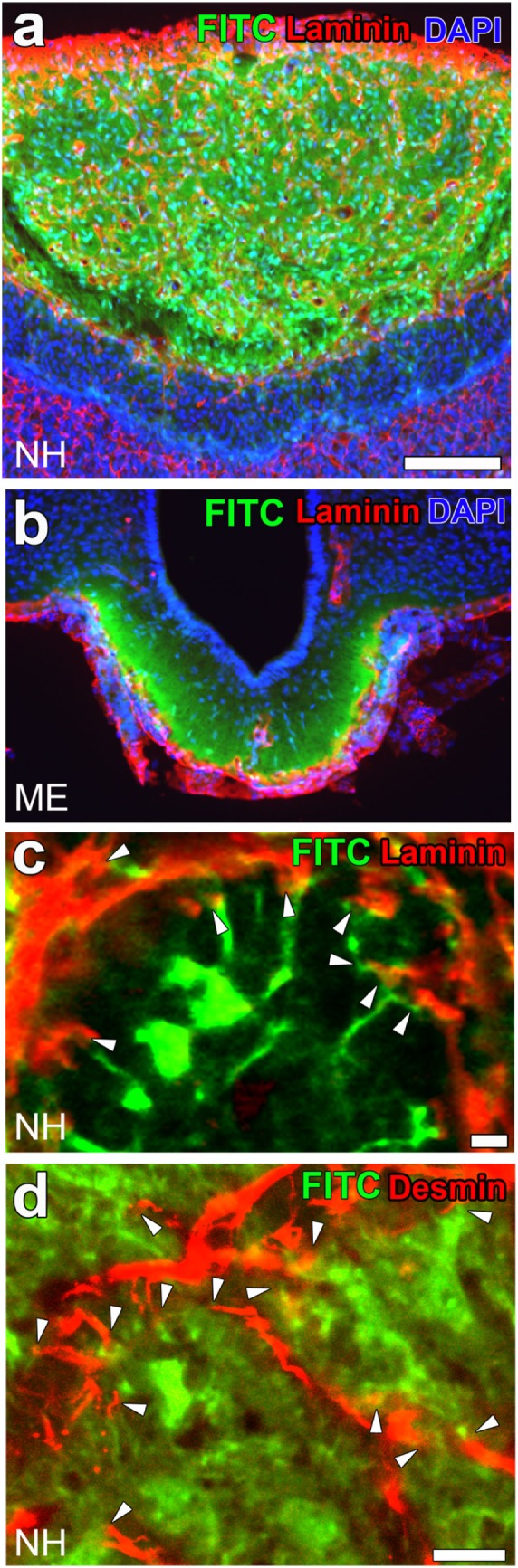
High vascular permeability of fenestrated capillaries in the neurohypophysis (NH) and median eminence (ME). Low magnification views reveal the strong fluorescence of the blood-derived low-molecular-weight (LMW) fluorescent molecule fluorescein isothiocyanate (FITC) in the NH **(A)** and ME **(B)**. A high magnification view shows the strong fluorescence of FITC at the interstitial space close to perivascular protrusions [**(C)**; arrowheads]. The fluorescence of FITC is also closely associated with the cellular processes of pericytes [**(D)**; arrowheads]. Scale bars represent 50 μm **(A)** and 10 μm **(C,D)**. Confocal images are rearranged with permission from Springer [**(A,B)** (28)] and John Wiley & Sons [**(C,D)** (23)].

The perivascular structures of the NH (47–50) and ME (51) largely differ from those generally observed in the brain because they have a wide perivascular space between the inner and outer BMs. Therefore, the profile of the outer BM or vascular surface in the NH [(40); Figure 2A] and ME [(42); Figure 2B] is more complex or uneven than that of the general BBB-containing brain vasculature [(52); Figure 2C]. This irregularity in the vascular surface is primarily due to the complex cellular processes of pericytes that function as vascular mural cells surrounding ECs [(23); Figure 2D]. In the NH, pericytes align parallel to ECs and extend their cellular processes or “perivascular protrusions” into the interstitial space between axon terminals [(23); Figure 2E]. Although the functional significance of a wide perivascular space or perivascular protrusions in neurosecretory regions currently remains unclear, a recent study indicated that this specialized structure is important for neurosecretion (23). The complex profile of the perivascular space largely contributes to increases in the vascular surface (23). Although the vascular permeability of LMW molecules is evidently higher in the NH and ME than in other brain regions and sensory CVOs [(28); Figures 3A,B], detailed analyses revealed that LMW molecules are more likely to diffuse through perivascular protrusions rather than evenly from vascular surfaces [(23); Figure 3C]. The diffusion route of LMW molecules is frequently associated with the cellular processes of pericytes [(23); Figure 3D]. Furthermore, the intimate spatial relationship between perivascular protrusions and OXT- and AVP-containing axonal terminals may enable the efficient diffusion of neuropeptides. Electron microscopic observations showed that perivascular protrusions occasionally associate with inter-endothelial junctions, suggesting that perivascular protrusions are formed at inter-endothelial junctions only (23). In the adenohypophysis, a time-limiting step analysis demonstrated that Dextran 20,000, corresponding to the size of GH, moved rapidly to the perivascular space from the interstitial space, and was then slowly cleared from the perivascular space (53). Collectively, these findings indicate that a complex and wide perivascular space and perivascular protrusions contribute to increasing contact between axonal terminals and the vascular BM, and also that perivascular protrusions are the main diffusion route for hypothalamic neuropeptides.

## Activity-Dependent Neuro-Glial Reorganization

The neural activities of hypothalamic OXT- and AVP-containing neurons in the SON and PVN are facilitated as the secretion of neurohypophysial neuropeptides is enhanced in response to a physiological stimulation. In the SON and PVN, structural reorganization is accompanied by the hypertrophy of somata, the formation of multiple synapses of afferent inputs, and an increase in the direct neuronal membrane apposition of somata and dendritic bundling by the retraction of astrocytic cellular processes during a chronic physiological stimulation (48, 56–59). This somatic and dendritic structural reorganization is considered to be associated with coordinate population activity in order to respond appropriately to altered physiological conditions (60). In addition to hypothalamic nuclei, structural reorganization is known to occur in the NH following a chronic physiological stimulation (48), which may lead to an increase in the diffusion efficiency of neurohypophysial neuropeptides. For example, a chronic osmotic stimulation increased the vascular permeability of FITC (23) and the small neutral amino acid, alpha-aminoisobutyric acid (27) in the NH.

**Figure 4:**
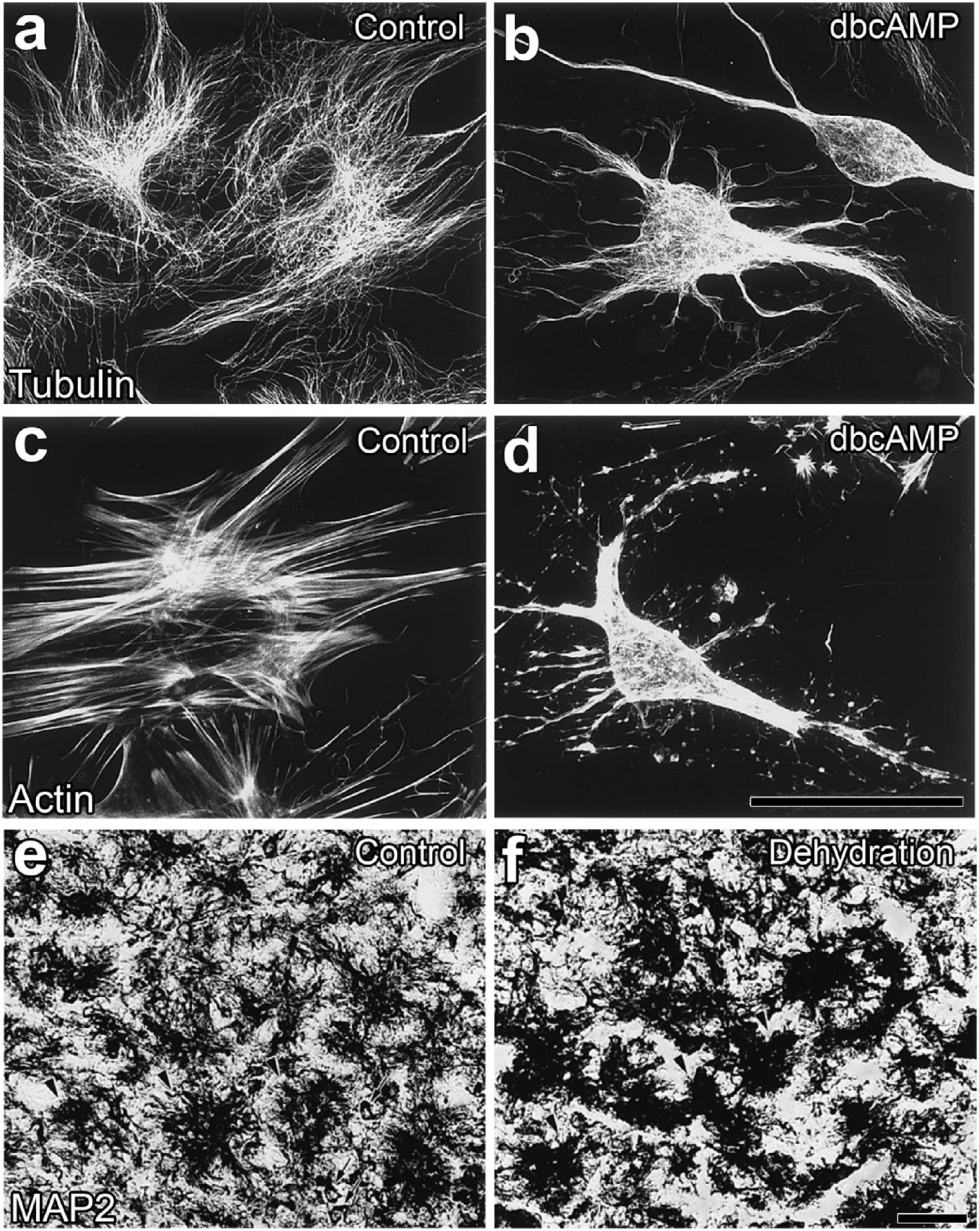
Morphological alterations in neurohypophysial glial cell pituicytes from a flat, polygonal shape to a stellate shape. Numerous fine fibers of microtubules are observed in flat pituicytes cultured from an explant of the rat neurohypophysis (NH), whereas microtubule fibers assemble in stellate pituicytes 60 min after a treatment with 1 mM dBcAMP **(A,B)**. The actin cytoskeleton is reorganized from normally occurring stress fibers into more diffusible actin upon a treatment with 1 mM dBcAMP for 60 min **(C,D)**. Microtubule-associated protein2-positive pituicytes of the NH of adult rats show a dendritic shape in unstimulated control animals, while their morphology becomes a less branched and aggregated shape upon a chronic osmotic stimulation **(E,F)**. Scale bars represent 50 µm. Micrographs are rearranged with permission from Elsevier [**(A–D)** (54)]; [**(E,F)** (55)].

A chronic physiological stimulation, such as hyperosmotic conditions as well as the suckling stimulation during lactation, has been shown to cause neuro-glial reorganization in the NH of adult rodents (48). Neurohypophysial glial cells, pituicytes, generally enclose the axonal terminals of magnocellular neurons and intervene between axonal terminals and the vascular BM under unstimulated conditions, while a chronic physiological stimulation increases the direct contact of axonal terminals to the vascular BM (47, 48, 61, 62). This neurovascular reorganization was previously considered to be caused by a shape conversion or the retraction of the cellular processes of pituicytes *via* β-adrenergic or adenosine receptors in cultured pituicytes [(54); Figures 4A–D] or isolated NH (63). Moreover, a similar shape conversion of pituicytes was demonstrated in animals that received a chronic hyperosmotic stimulation [(55); Figures 4E,F]. The stellation of pituicytes results from the inhibition of the small GTPase, RhoA and subsequent actin depolymerization (64–66). AVP and OXT have the ability to reverse the stellation of pituicytes and return them to their original shape by activating Cdc42, another small GTPase that reorganizes the actin cytoskeleton in a cortical position (65–67). The complex of dystrophins and dystrophin-associated-protein expressed on pituicytes interacts with laminin and the extracellular matrix, which may play a role in the retraction and reinsertion of the cellular processes of pituicytes during neuro-glial reorganization (68).

Tyrosine hydroxylase, the first rate-limiting enzyme of catecholamine synthesis, is localized at an extensive number of axonal terminals in the NH (69); however, its localization is restricted to the Herring bodies of AVP-containing axonal fibers (70). A chronic hyperosmotic stimulation has been shown to decrease AVP levels in the NH and increases those of tyrosine hydroxylase (70). ATP is present in millimolar concentrations in axon terminals and is co-released with neuropeptides in the NH (71). ATP is broken down to the metabolite product, adenosine at the extracellular space, and adenosine then acts on adenosine receptors (72). Collectively, these findings indicate that the activity-dependent release of noradrenaline and/or ATP from axonal terminals causes a shape conversion of pituicytes in the NH.

In the ME, neuro-glial reorganization similarly occurs in a manner that is dependent on increased demands for the secretion of adenohypophyseal hormones. The axonal terminals of GnRH neurons are separated from the BM of capillaries by the intervening cellular processes of tanycytes in the ME (73). Radial glial cells have been reported to give rise to mature ependymal cells as well as neurons and glial cells during development (74) and remain in existence at discrete regions of the adult central nervous system (75, 76). Residual radial glial cells, called tanycytes, are found lining the floor and ventrolateral walls of the third ventricle of the adult brain (75, 76). More axonal terminals of GnRH neurons contact the capillary BM in the estrogen high stage during proestrus than in the estrogen low stage during diestrus in rodents (77). During the preovulatory gonadotrophin surge, the retraction of tanycytic cellular processes enables the axonal terminals of GnRH neurons to directly contact the capillary BM through the estrogen-dependent secretion of nitric oxide (78–80). The axonal terminals of other types of adenohypophysial hormone-releasing hormones have not exhibited the neuro-glial specialization observed in GnRH neurons (81). The axonal terminals of GnRH neurons are often enwrapped by the cellular processes of tanycytes and sometimes localize in close proximity to fenestrated portal capillaries in the human ME (82). The secretion of GnRH was previously shown to be downregulated along with reductions in neurovascular contacts in aged rodents (83, 84) and humans (85). The sex steroid hormone estrogen induces the sudden and massive retraction of tanycyte cellular processes by inducing the release of nitric oxide from ECs (86, 87). Semaphorin7A, which is expressed by tanycytes, not only induces the retraction of GnRH axonal terminals but also promotes their ensheathment by tanycytic cellular processes through PlexinC1 and Itgb1 receptors (88). PlexinC1-deficient mice exhibit an increased density of GnRH axonal terminals in the ME and have an abnormal ovulation and estrous cycle, and tanycyte-specific Itgb1 silencing has been shown to promote the retraction of tanycyte cellular processes in order to increase neurovascular contact between the axonal terminals of GnRH neurons and the capillary BM (88). Neuro-glial plasticity in the ME has already been described in detail (80, 81, 11). Therefore, a shape conversion of glial cells occurs in the NH and ME in response to increased demands for neurosecretion in order for more hypothalamic axonal terminals to contact vascular surfaces.

## Pericyte-Dependent Reorganization of the Perivascular Space

**Figure 5:**
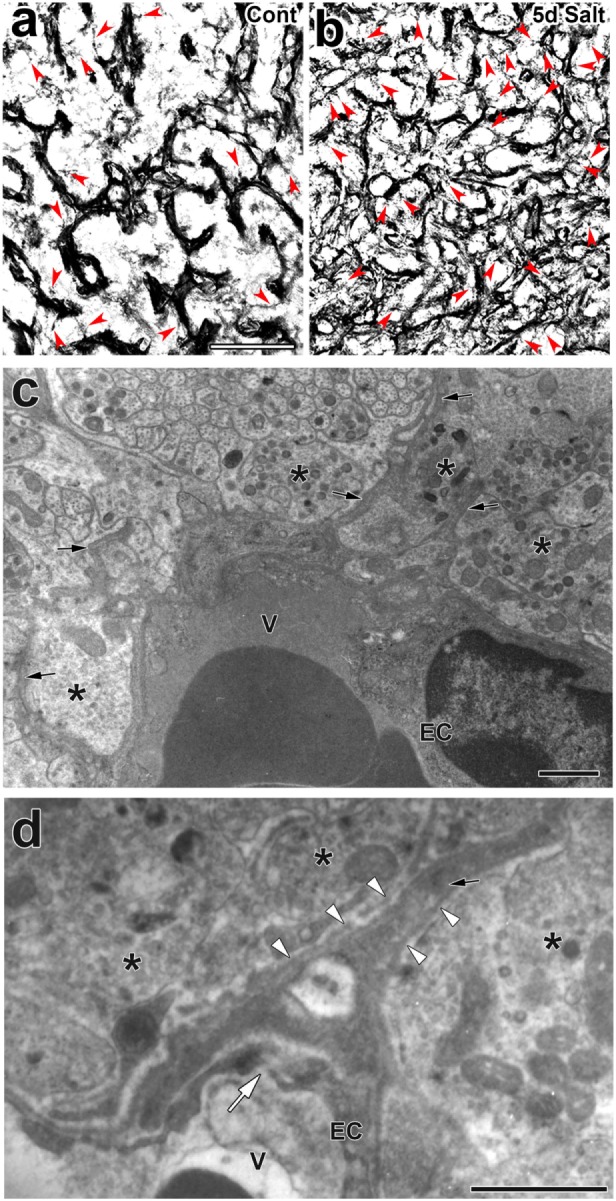
Activity-dependent morphological alterations in vascular mural cell pericytes in the adult mouse neurohypophysis (NH) by a chronic osmotic stimulation. Pericytes densely attach to the EC layer in order to constitute a thick mural cell layer under unstimulated control conditions, whereas they are likely to divide and extend their cellular processes upon a chronic osmotic stimulation [red arrowheads; **(A,B)**]. An electron micrograph reveals that a chronic osmotic stimulation changes the ultrastructure of the perivascular space with an increase in perivascular protrusions (solid arrows) in the adult mouse NH **(C)**. High magnification view of an electron micrograph showing that a perivascular protrusion (solid arrow) is enveloped by the outer basement membrane (open arrowheads) and associated with inter-endothelial junctions (open arrow) **(D)**. Asterisks indicate axonal terminals. Scale bars represent 50 μm **(A)** and 1 μm **(C)**. EC, endothelial cell; V, vascular lumen. Micrographs are rearranged with permission from John Wiley & Sons (23).

Activity-dependent neurovascular structural reorganization has been attributed to a shape conversion of glial cells until recently. Therefore, changes in vascular/perivascular structures were not investigated because the vascular system itself was considered to be unchanged. Pericytes are vascular contractile mural cells that form an incomplete layer on the abluminal side of the EC layer (89). They participate in modifying the vascular ultrastructure and gene expression of ECs in response to changes in the brain microenvironment (90). Pericytes have been shown to possess contractile properties *via* receptors for vasoactive molecules, such as catecholamine, endothelin-1, AVP, and angiotensin II (91). Recent studies demonstrated that pericytes are important for maintaining the tightness of the BBB and vascular density during adulthood as well as the formation of the BBB during development (15, 20, 21). We previously demonstrated a shape conversion of pericytes and structural changes in the perivascular space in the NH (23). Light microscopic analyses revealed that pericytes have a thick wall that wraps around the EC layer under unstimulated control conditions, but may develop thin cellular processes and bodies and extend these cellular processes to increase protrusions following a chronic osmotic stimulation [(23); Figures 5A,B]. A chronic hyperosmotic stimulation was shown to increase the number of pericyte cellular processes to 2.72-fold that of the control without changing the density of pericytes (23). Electron microscopic observations also revealed that a chronic osmotic stimulation caused pericytes to extend their cellular processes into the extracellular space between axonal terminals, thereby increasing the number of perivascular protrusions [(23); Figures 5C,D]. This perivascular reorganization is not accompanied by changes in the area of the perivascular space or ECs or the diameter of vessels.

**Figure 6:**
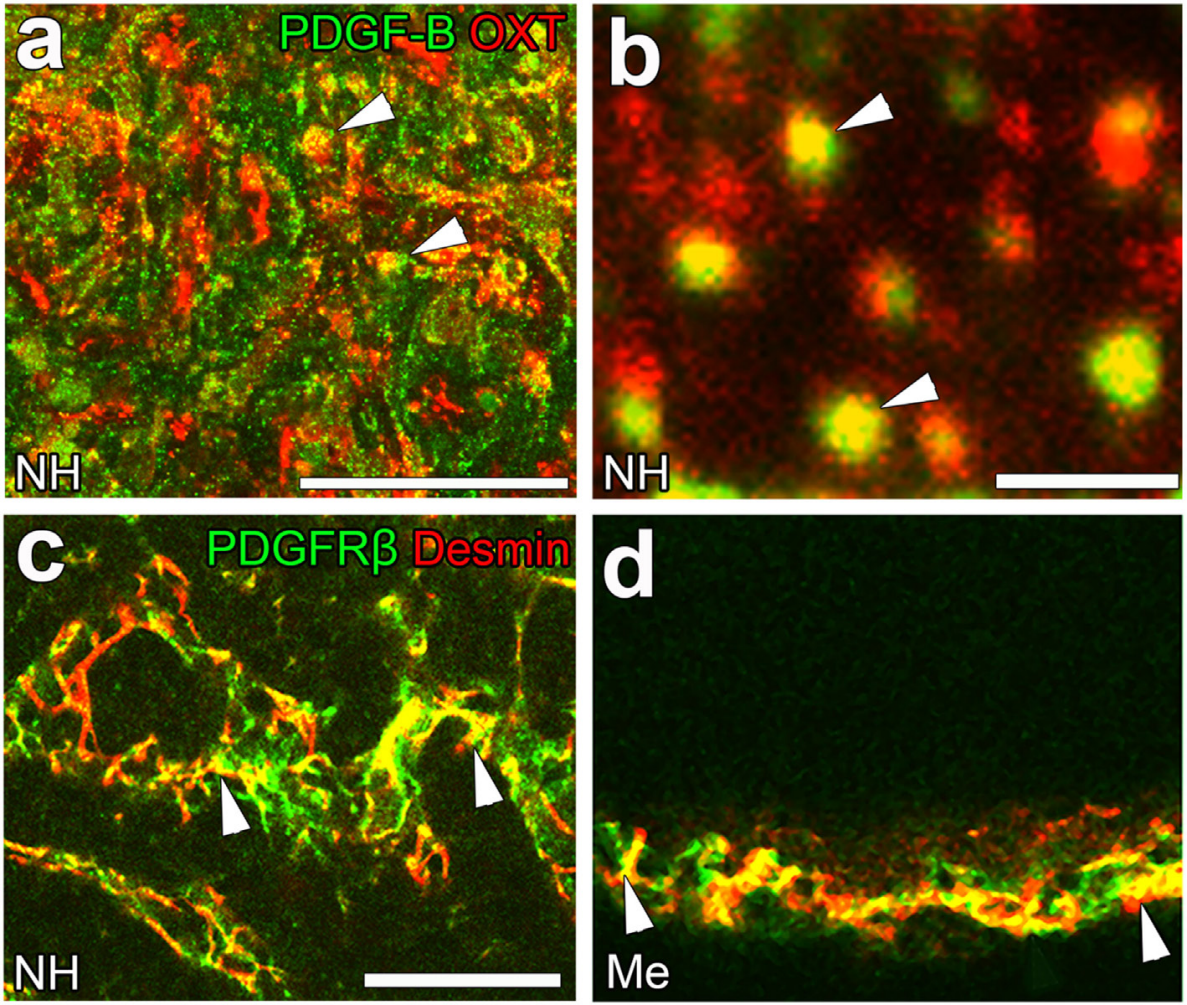
The expression of platelet-derived growth factor-B (PDGF-B) and PDGF receptor β (PDGFRβ) in the neurohypophysis (NH) and median eminence (ME) of adult mice. PDGF-B is prominently expressed at oxytocin (OXT)-containing axonal terminals [arrowheads, **(A)**] in the NH of the adult mouse. A high magnification view shows that PDGF-B is localized at neurosecretory granules within OXT-containing axonal terminals [arrowheads, **(B)**]. The strong expression of PDGFRβ is observed at desmin-positive pericytes in the NH [arrowheads, **(C)**] and ME [arrowheads, **(D)**]. Scale bars represent 50 μm **(A)** and 1 μm **(C)**. Me, ME; NH, NH. Confocal images are rearranged with courtesy from BioScientifica [**(A–C)** (40)] and permission from John Wiley & Sons [**(D)** (42)].

Although the signaling pathways that control the shape conversion of pericytes have not yet been elucidated in detail, the pericyte-regulating factor, platelet-derived growth factor-B (PDGF-B), is the most probable candidate. PDGF-B is known to be a requisite proliferative and chemoattractant factor for pericytes and mediates paracrine interactions between ECs and pericytes (92). A PDGF-B concentration gradient is necessary for pericyte attachment toward ECs as well as proliferation and migration (20). In the NH, PDGF-B is stored at neurosecretory granules in OXT-containing axon terminals [(23); Figures 6A,B]; however, PDGF-B is also expressed in ECs at the BBB (92). Moreover, the strong expression of PDGF receptor β (PDGFRβ) has been detected at pericytes in the NH [(23); Figure 6C] and ME [(42); Figure 6D]. These findings indicate that a dynamic shape conversion of pericytes causes the structural reorganization of the perivascular space with increases in perivascular protrusions, which, in turn, enlarge the terminal-contactable vascular area. Thus, activity-dependent neurovascular reorganization is caused by a shape conversion of both glial cell pituicytes and vascular mural cell pericytes.

## Changes in the EC Population by Angiogenesis

**Figure 7:**
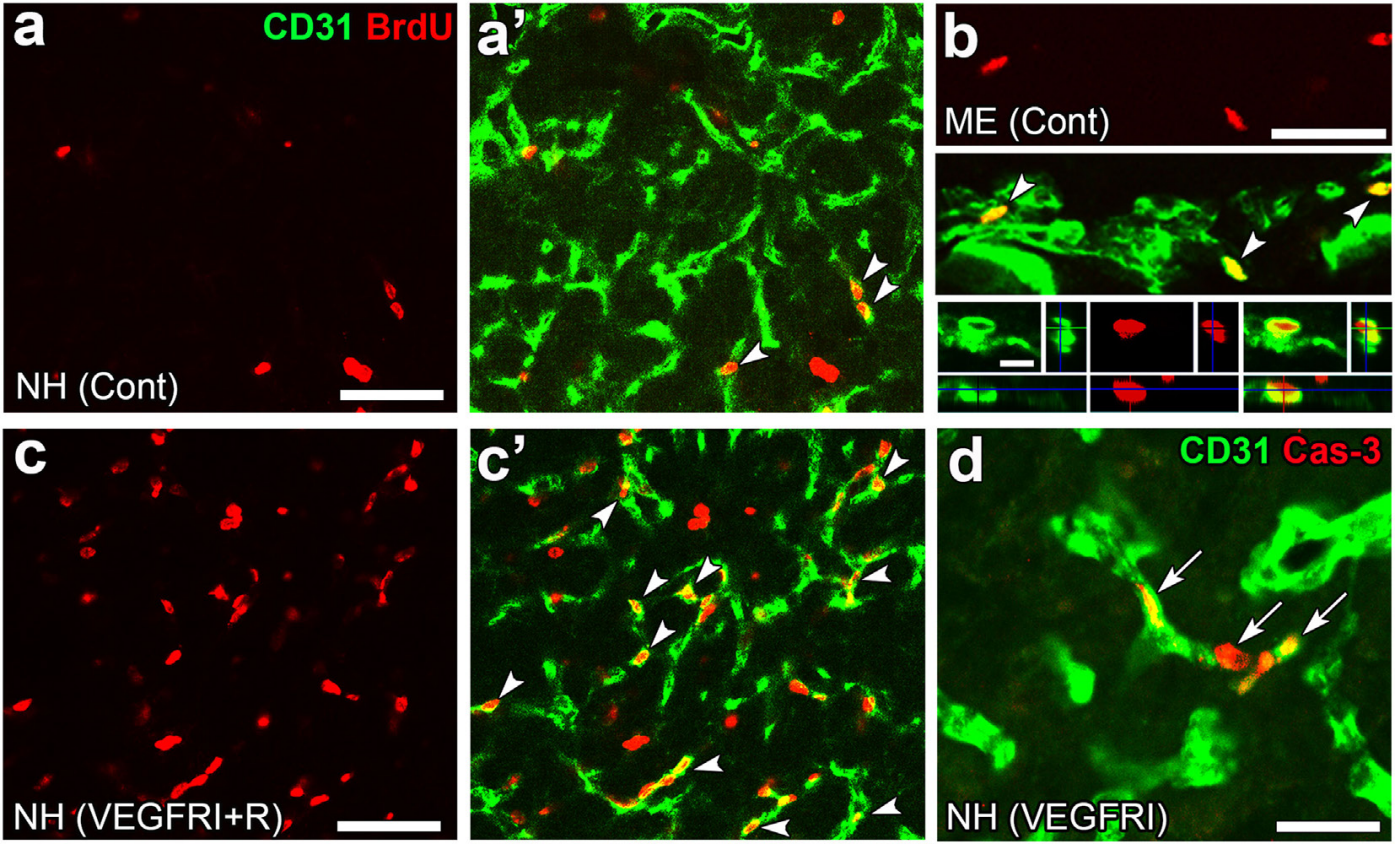
Continuous angiogenesis with the proliferation and apoptosis of endothelial cells (ECs) in the neurohypophysis (NH) and median eminence (ME) of adult mice. Double labeling immunohistochemistry shows the occurrence of BrdU-labeled nuclei (arrowheads) at ECs in the NH **(A,A’)** and ME **(B)**. The prominent rebound proliferation of ECs (arrowheads) in the NH after the withdrawal of a treatment with a VEGFR-associated tyrosine kinase inhibitor **(C,C’)**. The treatment with the VEGFR-associated tyrosine kinase inhibitor induces caspase-3-positive apoptotic ECs in the NH [arrows, **(D)**]. Scale bars represent 50 μm **(A–D)** and 5 μm [bottom panel in **(B)**]. BrdU, bromodeoxyuridine; Cas-3, caspase-3; Cont, control; Me, ME; NH, NH; VEGFRI, VEGFR-associated tyrosine kinase inhibitor; VEGFRI + I, VEGFRI plus a 5-day recovery period. Photographs are rearranged with courtesy from Bioscientifica [**(A–C,C’)** (40)] and permission from John Wiley & Sons [**(D)** (42)].

**Figure 8:**
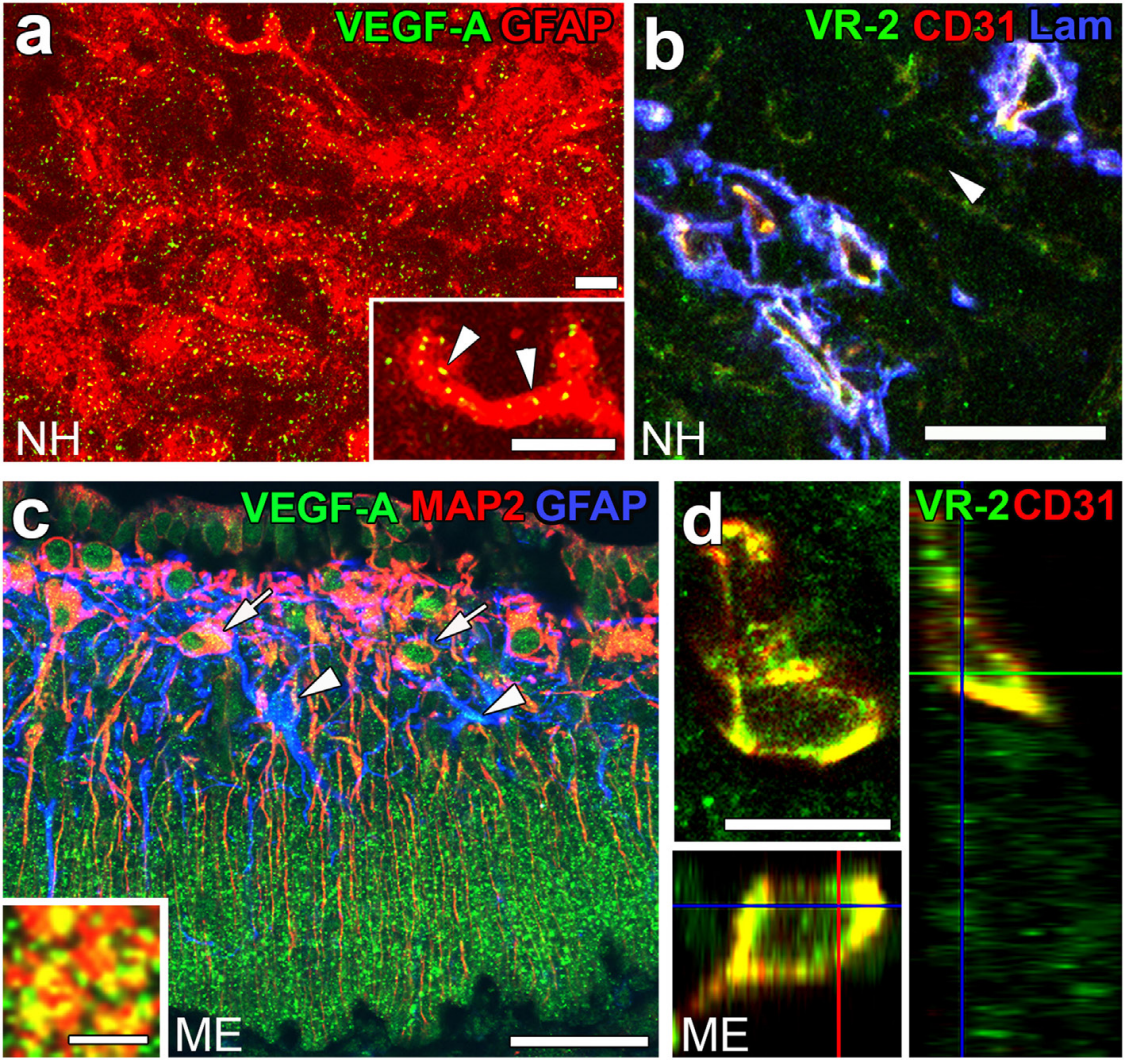
The expression of angiogenic factors vascular endothelial growth factor-A (VEGF-A) and VEGF receptor 2 (VEGFR2) in the neurohypophysis (NH) and median eminence (ME) of adult mice. VEGF-A and its receptor VEGFR2 are expressed at GFAP-positive astrocytes and CD31-positive endothelial cells (ECs), respectively **(A,B)**. VEGF-A is expressed at MAP2-positive somatodendrites and GFAP-positive astrocytes in the internal zone of the ME **(C)**. Inset indicates the presence of VEGF-A at synaptophysin-positive axonal terminals in the external zone of the ME. The expression of VEGFR2 is prominent at CD31-positive ECs in the ME **(D)**. Scale bars represent 50 μm **(A–C)**, 10 μm [**(D)** and inset in **(A)**], and 5 μm [inset in **(C)**]. GFAP, glial fibrillar acidic protein; Lam, laminin; MAP2, microtubule-associated protein 2; VR2, VEGFR2. Images are rearranged with courtesy from Bioscientifica [**(A,B)** (40)] and permission from John Wiley & Sons [**(C,D)** (42)].

**Figure 9:**
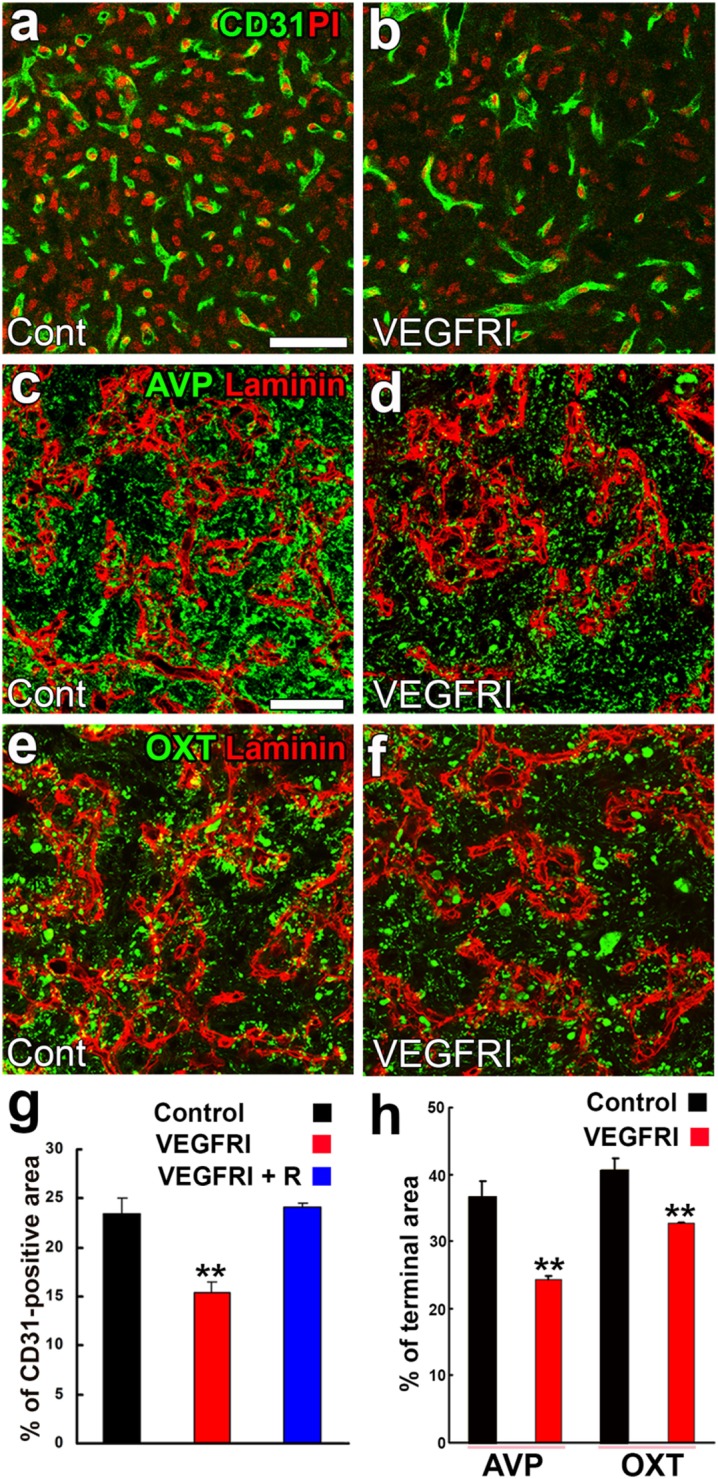
Effects of a VEGFR inhibitor on the density of endothelial cells (ECs) and axonal terminals in the neurohypophysis of adult mice. The VEGFR inhibitor AZD2171 significantly reduces the number of CD31-positive ECs **(A,B,G)**. The VEGFR inhibitor also diminishes the area of arginine vasopressin (AVP)- **(C,D,H)** and oxytocin (OXT)-containing **(E,F,H)** axonal terminals. Scale bars represent 50 µm. PI, propidium iodide; VEGFRI, VEGFR-associated tyrosine kinase inhibitor; VEGFRI + I, VEGFRI plus a 5-day recovery period. Data are rearranged with permission from BioScientifica (40).

Angiogenesis in the brain starts during the embryonic period and persists until the early postnatal period, but becomes completely quiescent with brain maturation (93, 94). The findings of our recent studies challenge this traditional concept that the density of ECs in adults remains unchanged throughout life. During angiogenesis, endothelial tip cells lead sprouting vessels, extend filopodia, and migrate in response to gradients of VEGF-A, while adjacent endothelial stalk cells trail tip cells and generate the trunks of new vessels (95). VEGF-A and its receptor VEGF receptor 2 (VEGFR2) are predominant angiogenic signaling molecules that control the proliferation and sprouting of ECs (95, 96). A large number of proliferating ECs have been detected in the NH [(40); Figures 7A,A’] and ME [(42); Figure 7B], even in healthy normal adult mice. A treatment with a VEGFR signaling inhibitor was found to significantly decrease the proliferation of ECs in the NH (40) and ME (42). After the cessation of this VEGFR inhibitor treatment, a marked increase was observed in the proliferation of ECs in the NH [(40); Figures 7C,C’]. By contrast, the VEGFR signaling inhibitor promoted the apoptosis of ECs in the NH [(40); Figure 7D]. The expression levels of VEGF-A are reported to be higher in the NH than in the adenohypophysis and are undetectable in the intermediate lobe (97). The expression of VEGF-A and VEGFR2 has been observed at pituicytes and ECs in the NH, respectively [(40); Figures 8A,B]. AVP and OXT both induce Ca^2+^ signals in pituicytes *via* the V1a subtype of AVP receptors in a manner that depends on extracellular Ca^2+^ (98–100). Glial cells secrete numerous transmitters and/or growth factors by Ca^2+^-dependent exocytosis (101). A chronic treatment with the VEGFR signaling inhibitor was shown to significantly decrease the area and density [(40); Figures 9A,B,G] of ECs, but did not significantly affect the area of ECs in the adenohypophysis, cortex, or peripheral tissues (40). In the ME, VEGF-A expression is prominent at axonal terminals and VEGFR2 is strongly expressed at ECs [(42); Figures 8C,D]. The peripheral and central administration of VEGF-A increases the density of PV-1-expressing fenestrated capillaries in the ME without affecting the expression of tight junction proteins (26). Thus, these recent findings demonstrate the presence of continuous angiogenesis in the NH and ME of adult rodents in a manner that is dependent on VEGF signaling.

A synchronized decrease has been observed in the densities of AVP- and OXT-containing axonal terminals and ECs following a chronic treatment with a VEGFR signaling inhibitor [(40); Figures 9C–F,H]. Microglia have been shown to engulf the axonal terminals of OXT- or AVP-containing neurons; some phagosomes and secondary lysosomes possess morphologically intact neurosecretory granules and others contain partially destroyed neurosecretory granules or amorphous material (102). This finding indicates that microglia are responsible for the remodeling of the axonal terminal arborization of neurosecretory neurons. Microtubule-associated protein-1B is strongly expressed at the sprouting axons and growth cones of developing or regenerating neurons and plays a role in axonal outgrowth (103). The expression level of phosphorylated microtubule-associated protein-1B was found to be markedly increased at axonal terminals in the NH during lactation (104). Thus, the population of axonal terminals is coordinately regulated to match that of ECs in adult rodents.

## Control of the Glial Population

As described above, pituicytes are resident glial cells in the NH and are responsible for neuro-glial structural reorganization. In explant cultures of newborn rat NH, oligodendrocyte progenitor cells differentiated into stellate-shaped type 2 astrocytes in the presence of serum, but developed into oligodendrocytes in its absence (105). Oligodendrocyte progenitor cells are known to be present and give rise to differentiated pituicytes in the NH of adult rodents (106). Moreover, a chronic osmotic stimulation promoted the proliferation of oligodendrocyte progenitor cells and increased the population of pituicytes (107). A PDGFR inhibitor was shown to significantly inhibit the proliferation of oligodendrocyte progenitor cells in the NH of adults (40). A previous study reported that PDGF-A promoted the proliferation, survival, migration, and differentiation of oligodendrocyte progenitor cells in the subventricular zone (108, 109). Although the functional significance of activity-dependent increases in the pituicyte population remains unknown, lactate transport between astrocytes and neurons is necessary for maintaining fully functional excitatory transmission between primary afferents and solitary neurons, even in the presence of a sufficient glucose supply (110). Thus, an increase in the population of pituicytes may result in the supply of more lactate to neurosecretory axonal terminals as an energy source for neurosecretion.

Oligodendrogenesis continuously occurs in adult brains throughout life (111). Oligodendrocyte progenitor cells originating in the neonatal SVZ have the ability to migrate into the white matter and cortex with widespread rostrocaudal dispersion (112, 113). In contrast to neonatal brains, the migration of oligodendrocyte progenitor cells in the adult SVZ is restricted to the corpus callosum, striatum, and fimbria fornix (114). All of the oligodendrocyte progenitor cells of adult brains retain their proliferative ability and are capable of restoring their population after a widespread loss (115). Sox2-expressing stem cells exist in a marginal zone of the anterior and intermediate pituitary lining the pituitary cleft and have the ability to form “pituispheres” in cultures that differentiate into all pituitary hormone-producing lineage cells (116, 117). These Sox2-expressing stem cells in adult pituitary glands contribute to pituitary homeostasis even 1 year after birth and, thus, are long-lived stem cells with the ability to generate fully differentiated hormone-producing cells throughout life, in contrast to short-lived progenitor cells (118, 119). Moreover, nestin-expressing Sox2-positive cells exist at the marginal zone and nestin-expressing Sox2-negative cells at the submarginal zone and body of the gland (117). The pituitary gland is entirely ectodermal in origin; the neural ectoderm gives rise to the NH and the adenohypophysis develops from Rathke’s pouch (120). We previously observed that GFP-expressing glial cells were present in the NH using Nestin-CreERT2 transgenic mice (unpublished data). Therefore, we cannot deny the possibility that neurohypophysial oligodendrocyte progenitor cells originate from nestin-expressing stem cells that exist in a marginal zone of the anterior and intermediate pituitary. Taken together, the pituicyte population in the NH is regulated by the proliferation of oligodendrocyte progenitor cells in a coordinated manner with ECs and axonal terminals.

Tanycytes are potential neural stem cells in the basal hypothalamus of mice (121). Tanycytes are present at the floor and ventrolateral walls of the third ventricle and are distinguished from ependymal cells by the presence of long radial cellular processes and lack of beating cilia (75, 76). α-Tanycytes located at the ventrolateral walls of the third ventricle extend cellular processes toward the arcuate and ventromedial hypothalamic nuclei, while β-tanycytes lining the floor of the third ventricle project cellular processes toward fenestrated capillaries in the ME (75). Lineage tracing using Nestin-CreERT2 revealed that β-tanycytes are the most proliferative among tanycytes in infant animals (122, 123). However, only α-tanycytes are neural stem/progenitor cells in the adult mouse because they were found to self-renew and give rise to new tanycytes, astrocytes, and small numbers of neurons in GLAST-CreERT2 transgenic mice (124). A lineage-tracing analysis using GLAST-CreERT2 transgenic mice showed that GFP expression was only detected in α-tanycytes, not β-tanycytes 5 days after tamoxifen-induced recombination and was increased in β-tanycytes after longer-term chase periods (124). Moreover, an *in vitro* analysis revealed that α-tanycytes, not β-tanycytes, proliferated in response to FGF-2 and possessed neurosphere-forming and self-renewal abilities (124). Taken together, tanycytes may also proliferate during adulthood which is likely due to an intrinsic stem cell capacity as it is not clear if (1) a given tanycyte divides continuously during adulthood and (2) if many or only very few actually proliferate at all.

## Notch Signaling and the Extracellular Microenvironment

**Figure 10:**
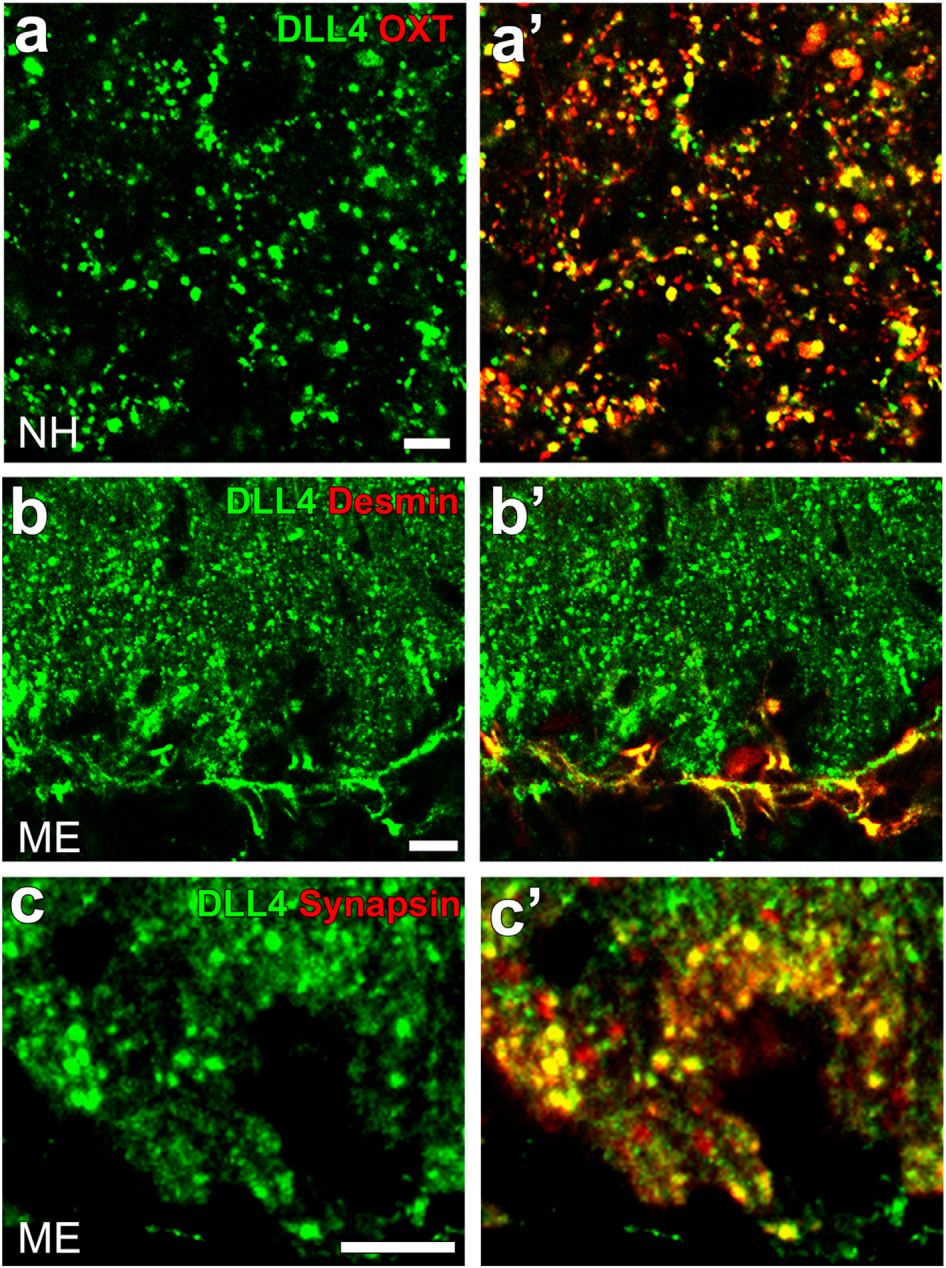
Confocal microscopic images showing the localization of DLL4 in the neurohypophysis (NH) and median eminence (ME) of an adult mouse. DLL4 expression is observed at oxytocin-containing axonal terminals in the NH **(A,A’)** and at desmin-positive pericytes **(B,B’)** and synapsin-positive axonal terminals **(C,C’)** in the ME. Scale bars represent 50 μm **(A)** and 10 μm **(B,C)**. Confocal micrographs are rearranged with permission from John Wiley & Sons [**(A,A’)** (41)], [**(B,B’)**; **(C,C’)** (42)].

Notch signaling occurs between neighboring cells that express Notch receptors and ligands (Jagged1 and 2 and DLL1, 3, and 4) and acts in cell-fate decisions and morphogenesis during development (125). Although most studies on Notch signaling in brains have been performed on the proliferation and differentiation of stem/progenitor cells in developing brains, recent studies have shown that Notch signaling controls synaptic plasticity and behavior. For example, Notch signaling or the cleavage of Notch1 is activated by an increase in synaptic activity and plays a role in synaptic plasticity in the hippocampal CA1 region (126). DLL4 and Notch3 were found to be expressed at OXT-containing axonal terminals and pituicytes in the NH, respectively [(41); Figures 10A,A’]. Similarly, the strong expression of DLL4 was observed at axonal terminals and vascular pericytes in the ME [(42); Figures 10B,B’,C,C’].

The binding of Notch and its ligand causes the proteolytic cleavage of Notch *via* presenilin protease of the c-secretase complex and the Notch intracellular domain, or a cleaved Notch form, then translocates to the nucleus in order to control the transcription of target genes, including neural cell adhesion molecule (NCAM), F3, and tenascin-C (127–129). Chronic and acute osmotic stimuli have been shown to significantly increase the cleavage of Notch3 in the NH (41). Previous studies reported that the adhesion molecules polysialic acid–NCAM, F3, and tenascin-C are possible candidates for neurovascular and/or neuro-glial reorganization in the NH (130, 131). Polysialic acid–NCAM was shown to be markedly decreased during lactation in the NH and returned to its initial level only after weaning, and a decline in polysialic acid on the NCAM has been suggested to stabilize newly established neurovascular contacts (104). The removal of polysialic acid from the NCAM with endo-N prevented the structural reorganization of GnRH axon terminals as well as surrounding tanycytes in the ME (132). Moreover, Notch1 signaling has been identified as an upstream regulator of PDGF-B expression in vascular ECs and plays a crucial role in BBB integrity (133). Thus, Notch signaling is a key factor that regulates a number of regulatory molecules in order to cause structural reorganization in the NH and ME.

In addition to cell adhesion molecules, many types of extracellular matrix proteins such as chondroitin sulfate proteoglycans are expressed in the NH (49). During the development and reorganization of tissue, matrix metalloprotease-3, which belongs to a family of zinc-binding endopeptidases, has been shown to degrade various extracellular matrix molecules, including fibronectin, collagen, and laminin (134) as well as phosphacan, neurocan, versican, brevican, and NG2 (135). Tissue plasminogen activator also has crucial functions in the plasticity and development of brains (136, 137). Matrix metalloprotease-3 and tissue plasminogen activator are strongly expressed at axonal terminals in the NH (50, 138) and ME (139). Plasminogen is also strongly expressed in the NH and ME (50, 140). The depolarizing agent KCl releases tissue plasminogen activator from isolated neurosecretosomes in a manner that depends on Ca^2+^ (138). The recombinant tissue plasminogen activator has the ability to promote the release of AVP from isolated NH (50). Moreover, tissue plasminogen activator- and plasminogen-deficient mice both showed a weaker ability to secrete AVP into the circulation upon a chronic osmotic stimulation (50). Thus, changes in the microenvironment by extracellular matrix degradation enzymes may be necessary for inducing structural reorganization in the NH and ME.

**Figure 11:**
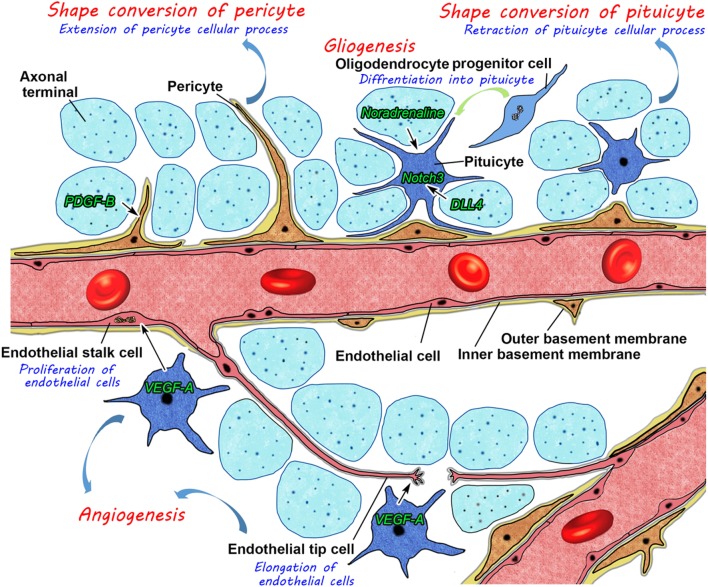
Schematic illustration showing possible mechanisms for neurovascular structural reorganization by a shape conversion of glial cell pituicytes and vascular mural cell pericytes and alterations in glial and endothelial cell populations by gliogenesis and angiogenesis, respectively, in the neurohypophysis of an adult mouse.

In summary, these findings have prompted us to propose a new model for structural reorganization in the NH (Figure 11). Glial cell pituicytes wrap around axonal terminals and interpose between axonal terminals and the BM of fenestrated capillaries under healthy normal conditions, and neurovascular structural reorganization is simply caused by the retraction of these cellular processes of pituicytes in response to a chronic physiological stimulation. This notion is based on the assumption that the vascular system remains unchanged in adult mammals. However, our recent findings disprove this notion and demonstrate the presence of dynamic changes in vascular/perivascular structures. A chronic osmotic stimulation causes a shape conversion of vascular mural cell pericytes, which results in an increase in perivascular protrusions and the vascular surface area. Moreover, we demonstrated the occurrence of the continuous proliferation of ECs in healthy adult rodents; however, angiogenesis is considered to occur during the embryonic and early postnatal periods and rarely during adulthood in the central nervous system. VEGF signaling coordinately regulates the population of ECs and axonal terminals. Glial cell pituicytes are continuously replaced by the differentiation of oligodendrocyte progenitor cells. This new model for structural reorganization in the NH may also be the case in the ME. Thus, recent evidence revealed that the hypothalamic–pituitary neurosecretory system has more dynamic and complicated mechanisms for structural reorganization than initially considered. These findings will set the groundwork for understanding hypothalamic neurosecretion and developing treatments for diseases of hypothalamic–pituitary neurosecretory dysfunctions and age-related decline in humans.

## Author Contributions

The author confirms being the sole contributor of this work and approved it for publication.

## Conflict of Interest Statement

The author declares that the research was conducted in the absence of any commercial or financial relationships that could be construed as a potential conflict of interest.
